# *In vitro* anthelmintic activity of an aqueous extract of *Glycyrrhiza glabra* and of glycyrrhetinic acid against gastrointestinal nematodes of small ruminants

**DOI:** 10.1051/parasite/2021060

**Published:** 2021-09-01

**Authors:** Michela Maestrini, Marcelo Beltrão Molento, Mario Forzan, Stefania Perrucci

**Affiliations:** 1 Department of Veterinary Sciences, University of Pisa Viale delle Piagge 2 56124 Pisa Italy; 2 Department of Veterinary Medicine, University of Paraná R. dos Funcionarios, 1540 Curitiba 80035-050 PR Brazil

**Keywords:** *Glycyrrhiza glabra*, Glycyrrhetinic acid, Gastrointestinal strongyles, Sheep, Anthelmintic activity, *In vitro*

## Abstract

This study evaluated the *in vitro* anthelmintic activity of a liquorice (*Glycyrrhiza glabra*) root aqueous extract and of glycyrrhetinic acid at 30, 10, 5, 1, and 0.5 mg/mL against sheep gastrointestinal nematodes (GINs), using the egg hatch test (EHT), the larval development test (LDT), and the larval migration inhibition test (LMIT). The compounds were applied on a mixture of GIN eggs and larvae, mainly *Trichostrongylus* spp. and *Teladorsagia/Ostertagia* spp. Cytotoxicity assays were also performed. In the EHT, both candidates showed significant concentration-dependent efficacy and were significantly more effective (*p* < 0.001) at the highest concentrations (30 and 10 mg/mL) than the lowest ones. In the LDT, only *G. glabra* showed a concentration-dependent effect (*R*^2^ = 0.924), but glycyrrhetinic acid (*R*^2^ = 0.910) had significantly higher efficacy than *G. glabra* root extract. Moreover, the efficacy of glycyrrhetinic acid at 30, 10, and 5 mg/mL was significantly higher (*p* < 0.001) than at lower concentrations. In the LMIT, *G. glabra* showed concentration-dependent efficacy (*R*^2^ = 0.971), while considerably reduced efficacy was observed for glycyrrhetinic acid (*R*^2^ = 0.855) at the lowest concentrations. These data suggest that the two compounds may have different mechanisms of action. In the LMIT, the 50% lethal concentration (LC_50_) of glycyrrhetinic acid (~5.12 mg/mL) was > 2.0-fold lower when compared to *G. glabra* (12.25 mg/mL). Analysis and previous findings indicated low toxicity for both compounds. The results obtained encourage *in vivo* studies aimed at evaluating the potential use of the tested compounds as natural de-wormers in ruminants.

## Abstract (fr)

Cette étude a évalué l’activité anthelminthique *in vitro* d’un extrait aqueux de racine de réglisse (*Glycyrrhiza glabra*) et de l’acide glycyrrhétinique à 30, 10, 5, 1 et 0,5 mg/mL contre les nématodes gastro-intestinaux (NGI) du mouton, en utilisant le test d’éclosion des œufs (TEO), le test de développement larvaire (TDL) et le test d’inhibition de la migration larvaire (TIML). Les composés ont été appliqués sur un mélange d’œufs et de larves de NGI, principalement *Trichostrongylus* spp. et *Teladorsagia/Ostertagia* spp. Des tests de cytotoxicité ont également été effectués. Dans le TEO, les deux candidats ont montré une efficacité concentration-dépendante significative et ont été significativement plus efficaces (*p* < 0,001) aux concentrations les plus élevées (30 et 10 mg/mL) qu’aux plus faibles. Dans le TDL, seul *G. glabra* a montré un effet concentration-dépendant (*R*^2^ = 0,924), mais l’acide glycyrrhétinique (*R*^2^ = 0,910) avait une efficacité significativement plus élevée que l’extrait de racine de *G. glabra*. De plus, l’efficacité de l’acide glycyrrhétinique à 30, 10 et 5 mg/mL était significativement plus élevée (*P* < 0,001) qu’à des concentrations plus faibles. Dans le TIML, *G. glabra* a montré une efficacité concentration-dépendante (*R*^2^ = 0,971), tandis qu’une forte réduction d’efficacité a été observée pour l’acide glycyrrhétinique (*R*^2^ = 0,855) aux concentrations les plus faibles. Ces données peuvent suggérer que les deux composés peuvent avoir des mécanismes d’action différents. Dans le TIML, la concentration létale à 50% (CL50) de l’acide glycyrrhétinique (~ 5,12 mg/mL) était > 2,0 fois inférieure à celle de *G. glabra* (12,25 mg/mL). L’analyse et les résultats précédents ont indiqué une faible toxicité pour les deux composés. Les résultats obtenus encouragent les études *in vivo* visant à évaluer l’utilisation potentielle des composés testés ici comme anthelminthique naturels chez les ruminants.

## Introduction

Infections caused by gastrointestinal nematodes (GIN) are considered one of the main causes of production losses, health problems, and poor welfare in small ruminants worldwide [51]. For more than 50 years, the control of GIN infections in livestock has relied mainly on the use of synthetic anthelmintic drugs [42]. However, in recent years a drastic reduction of the effectiveness of these drugs has been observed, caused by the selection of anthelmintic-resistant parasite populations, especially in sheep and goats [24, 43]. In Europe, a further limitation to the use of anthelmintic drugs for the control of GIN is linked to organic breeding, in which the use of synthetic drugs is limited by European regulations [13, 14].

Currently, the search for alternative control methods of ovine GIN is considered an important and urgent issue [37, 50, 51]. Among these alternative strategies, there is considerable and expanding interest in the search for effective plant extracts and plant-derived active components, namely plant secondary metabolites [5, 37]. The latter include mainly tannins, terpenoids, saponins, and flavonoids, as these components have been related to effective responses *in vitro* and/or *in vivo* against GINs of small ruminants (i.e., *Haemonchus* spp., and *Trichostrongylus* spp.) in different countries [18, 19, 28, 33, 34, 36, 44, 45].

Previous studies have shown promising *in vitro* antiparasitic properties of plant species belonging to the genus *Glycyrrhiza*. The genus *Glycyrrhiza* (Fabaceae) consists of about 30 plant species, including the species *Glycyrrhiza glabra* and *Glycyrrhiza inflata* [41]. The crude extract of *G. inflata* roots, also known as Chinese liquorice [38, 39], was found to be effective against the promastigote and amastigote stages of *Leishmania* spp. [39]. Another interesting study carried out by Aleixo et al. [1], has demonstrated the ability of *G. inflata* to alter cell integrity causing the death of *Schistosoma mansoni*, also affecting its motility and fertility. Moreover, the anthelmintic activity of an ethanolic extract and, albeit with lower efficacy, also of an aqueous rhizome extract of *G. glabra* have demonstrated *in vitro* activity against adults of *Haemonchus contortus* collected from the abomasum of infected sheep, using a micro-motility assay [29].

*Glycyrrhiza glabra* is a flowering plant commonly known as liquorice, native to Mediterranean areas, but now present in India, Russia, and China [41]. Glycyrrhetinic acid, also known as 18β-glycyrrhetinic acid, glycyrrhetic acid or enoxolone, is a pentacyclic triterpenic organic acid considered to be the major active component of *G. glabra* root aqueous extract. *Glycyrrhiza glabra* root may contain 2 to 25% glycyrrhetinic acid, mainly as glycosidic glycyrrhizinic acid saponin [20], along with other compounds such as polyphenols, saponins, and triterpenes [20, 40]. Limited anthelmintic potential of glycyrrhetinic acid was assessed in a study by Kalani et al. [27], where the authors have shown that this compound and some of its synthetic derivatives were effective *in vitro* against microfilariae and the adult stage of *Brugia malayi*.

The aim of the present study was to evaluate the *in vitro* activity of an aqueous extract of *G. glabra* root and of glycyrrhetinic acid against GINs of small ruminants.

## Materials and methods

### Plant materials

A commercial lyophilized aqueous extract obtained from the roots of liquorice plant (*G. glabra*) containing 10% glycyrrhetinic acid, mainly as glycyrrhizinic acid saponin (EPO Instituto Farmochimico Fitoterapico Srl, Milan, Italy), was diluted in distilled water at the final tested concentrations (30, 10, 5, 1, and 0.5 mg/mL). Glycyrrhetinic acid used in this study was a commercial sample (18β-glycyrrhetinic acid, Sigma, Italy). Glycyrrhetinic acid was diluted 1:1 (w/v) in a mixture composed by 0.1 mL of DMSO/mL in distilled water, then further diluted at the above tested concentrations.

### GIN eggs and third stage larvae (L3) recovery, purification, and identification

Individual faecal samples were collected from the rectum of naturally infected ewes. Ewes had polyparasitism with different GIN genera and species. Parasitological analysis of the collected samples was performed using a McMaster method, with a sensitivity of 50 eggs per gram (EPG) of faeces [47]. Faecal samples scoring positive for at least 1000 EPG were pooled and used in the assays, and for preparing the faecal cultures to obtain fresh L3. L3 were identified to the genus level. Recovery, suspension, and cultivation of eggs were performed using a previously reported protocol [25], with small modifications. In short, 30 g faecal material was homogenised in distilled water, placed inside a 50 mL tube, and centrifuged for 5 min at 2300 rpm. The sediment was collected and suspended in saturated NaCl solution (specific density 1.2) and centrifuged for another 5 min at 1000 rpm. The supernatant was then collected, diluted in distilled water in 15 mL tubes and then centrifuged for 5 min at 800 rpm. The sediment containing the eggs was collected and diluted in 1 mL of distilled water for GIN eggs/mL determination. To obtain L3 from eggs, faecal cultures were performed with pooled positive samples. Copro-cultures were placed in an incubator at 27 °C from 7 to 10 days. L3 were recovered by the Baermann technique and used in the LMIT. Moreover, about 100 larvae were microscopically identified to the genus level, based on their morphological and metric features [49]. In brief, L3 identification was based on several ensheathed L3 characteristics, including L3 dimensions (length and width), number and shape of intestinal cells, length and shape of the tail, shape of the head, the presence or absence of cranial refractile spots, and length and shape of the oesophagus. The presence or absence of digitate appendages on the tail of exsheathed (2% hypochlorite- treated) L3 was also evaluated [49].

### In vitro tests: egg hatch test (EHT), larval development test (LDT), and larval migration inhibition test (LMIT)

The EHT was performed according to the method described by Coles et al. [8]. Using 24-well cell culture plates, 100 purified eggs were placed in each well with 1 mL of a solution containing different concentrations (30, 10, 5, 1, and 0.5 mg/mL) of the tested compounds. Plates were incubated at 26 °C in darkness and 80% humidity, checked after 48h under an inverted microscope. The number of unhatched and hatched eggs was calculated for each well.

In the LDT, motile L1 obtained from EHT control plates were used by placing about 100 L1/well containing 1 mL of a solution made with the tested concentrations in the culture medium. Each mL of the culture medium contained 0.54 mL of saline solution, 0.06 mL of Earl Balanced Salt Solution (*Escherichia coli* lyophilised cells of Strain W) (Sigma Aldrich Srl, Milan, Italy), 12 μL of *E. coli* lyophilised and sterilised for 1 h at 100 °C (Sigma Aldrich Srl, Milan, Italy), 12 μg of amphotericin B (Amphotericin B from *Streptomyces* approx. 80% HPLC, Sigma Aldrich, Milan, Italy), 60 μg of yeast extract (Sigma Aldrich, Milan, Italy) and 0.24 mL of distilled water. Plates were incubated at 26 °C in darkness and 80% humidity and checked after 7 days under an inverted microscope to evaluate L3 morphology [35, 49]. The percentage of L3 from the total number of larvae was calculated for each well.

The LMIT was based on Demeler et al. [11], with modifications. For the test, about 100 exsheathed (2% hypochlorite) motile L3 were placed in each well, containing 1 mL of the tested compounds. Plates with L3 were incubated at 26 °C in darkness and 80% humidity, and live L3 were allowed to swing through a 25 μm mesh. The meshes were removed after 24 h, and the L3 that had migrated, were present at the bottom of each well. The efficacy of each concentration on L3 was measured according to the formula:

E(%)=(Total number of L3-Number of migrated L3/Total number of L3)×100.

In all assays, the effects of liquorice extract and glycyrrhetinic acid were compared with untreated (1 mL of distilled water or 1 mL of a mixture composed by 0.1 mL of DMSO/mL of distilled water) and treated positive controls (0.1 mg/mL thiabendazole (2-(4-Thiazoly) Benzimidazole) (Sigma, Milan, Italy) (TBZ) diluted in distilled water, or in a mixture composed of 0.1 mL of DMSO/mL in distilled water. All assays were performed in three replicates for each concentration of examined compounds and controls.

### Cytotoxicity assay

The cytotoxicity of *G. glabra* aqueous extract was evaluated on Madin-Darby bovine kidney (MDBK) cells using the Cytotoxicity LDH Assay Kit-WST (Dojindo laboratories, Dojindo EU GmbH, Munich, Germany), following the manufacturer’s instructions. This is a commercial kit for the determination of cytotoxicity by measuring lactate dehydrogenase (LDH), which is a cytoplasmic enzyme present in all types of cells and released from damaged cells. LDH catalyses dehydrogenation of lactate to pyruvate reducing NAD to NADH. NADH reduces a water-soluble tetrazolium salt in the presence of an electron mediator to produce an orange formazan dye. The amount of formazan dye is proportional to released LDH into the medium, that is an indication of cytotoxicity. More specifically, cells were plated at 1.5 × 10^3^/well in 96 well tissue flat bottomed culture plates and incubated at 37 °C in a 5% CO_2_ incubator for 24 h. The cells were washed in Dulbecco’s Modified Eagle Medium without phenol red and incubated for 1 h at 37 °C in the presence of scalar concentrations (30, 10, 5, 1, and 0.5 mg/mL) of *G. glabra* root. Then, the lysis buffer was added, and the plates incubated again for 30 min at the same conditions as above. The absorbance was measured at 490 nm by a microplate reader (ThermoFisher Scientific, Rodano, Milan, Italy).

### Statistical analysis

Descriptive analysis was carried out to demonstrate the compounds’ effects. The One-Way Analysis of Variance, ANOVA, and *t*-test were used to determine the differences among concentrations at the 0.05 level, as the data were tested for normality. The coefficient of determination using Pearson (*R*^2^) was calculated for each compound and for each *in vitro* test. The latter test was used to demonstrate the associated fitted efficacy values. The lethal concentration of 50% (LC50) based on Probit transformation (log-1) was calculated to determine the differences among products in each test, using Prism 7.1 software.

## Results

**Figure 1:**
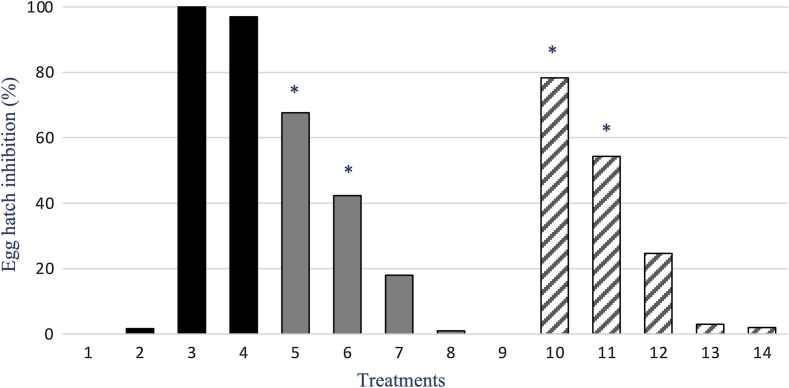
Percentage (%) of gastrointestinal strongyle egg hatch inhibition by five concentrations of *Glycyrrhiza glabra* root aqueous extract and of glycyrrhetinic acid. Columns: 1: Untreated, 2: DMSO, 3: Thiabendazole, 4: Thiabendazole plus DMSO, 5: 30 mg/mL, 6: 10 mg/mL, 7: 5 mg/mL, 8: 1 mg/mL, 9: 0.5 mg/mL of *G. glabra*, and 10: 30 mg/mL, 11: 10 mg/mL, 12: 5 mg/mL, 13: 1 mg/mL, 14: 0.5 mg/mL of glycyrrhetinic acid.

**Figure 2:**
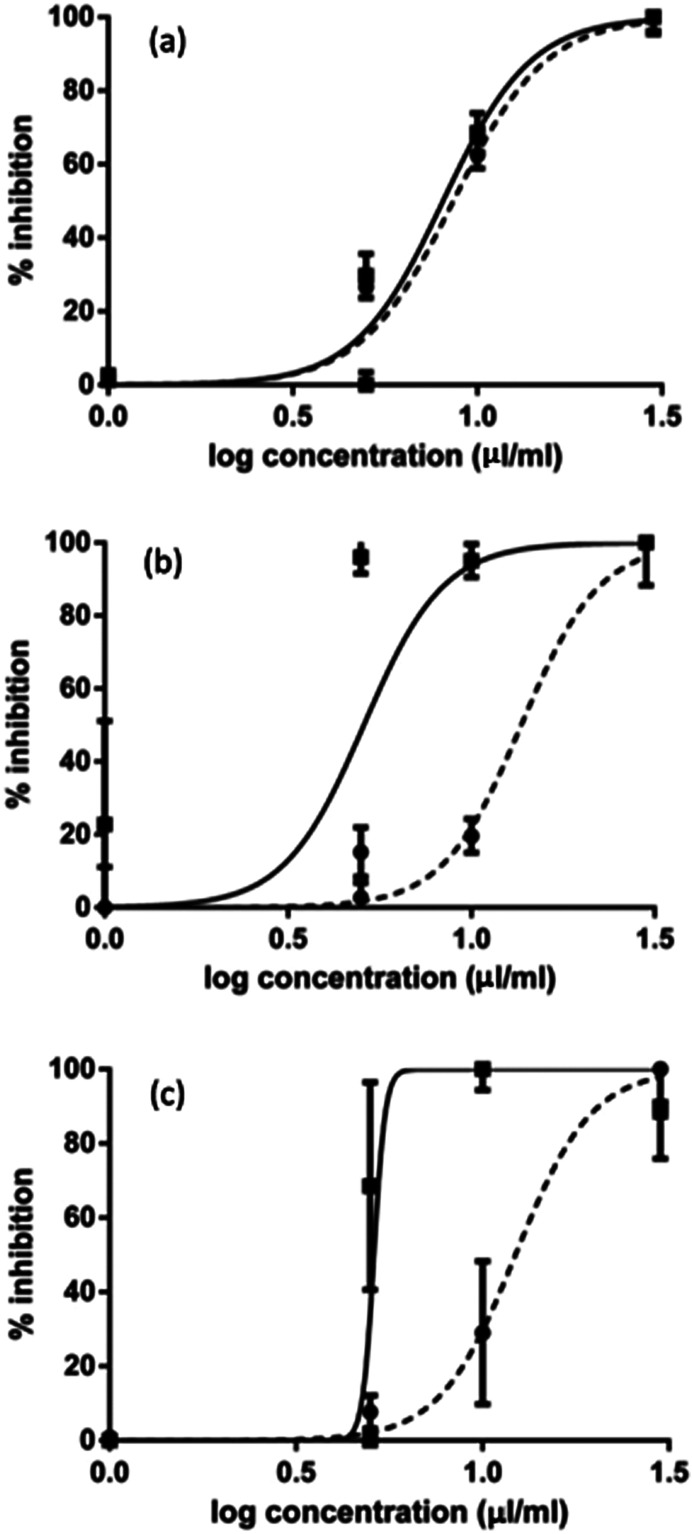
Efficacy (%) of different concentrations (log-1) (mg/mL) of *Glycyrrhiza glabra* root aqueous extract (dashed line) and of glycyrrhetinic acid (solid line) for the (a) egg hatch test (EHT), (b) > larval development test (LDT), and (c) larval migration inhibition test (LMIT) against gastrointestinal strongyles of sheep.

The results obtained with the EHT are shown in Figure 1. The untreated and the solvent DMSO control groups demonstrated no effect on eggs, and the TBZ and TBZ plus DMSO groups showed efficacies > 98%. Both candidate compounds showed a concentration-dependent anthelmintic effect with an *R*^2^ = 0.994 for *G. glabra* and *R*^2^ = 0.984 for glycyrrhetinic acid. For the two compounds, the efficacy of the higher concentrations (30 and 10 mg/mL) was significantly different (*p* < 0.001) from that of the lower concentrations. Glycyrrhetinic acid demonstrated better efficacy against egg development when compared to *G. glabra*, by an average of 10%. The LC_50_ was somewhat similar for both candidates (Fig. 2a), as the values were 8.61 and 8.07 mg/mL for *G. glabra* and glycyrrhetinic acid, respectively.

**Figure 3:**
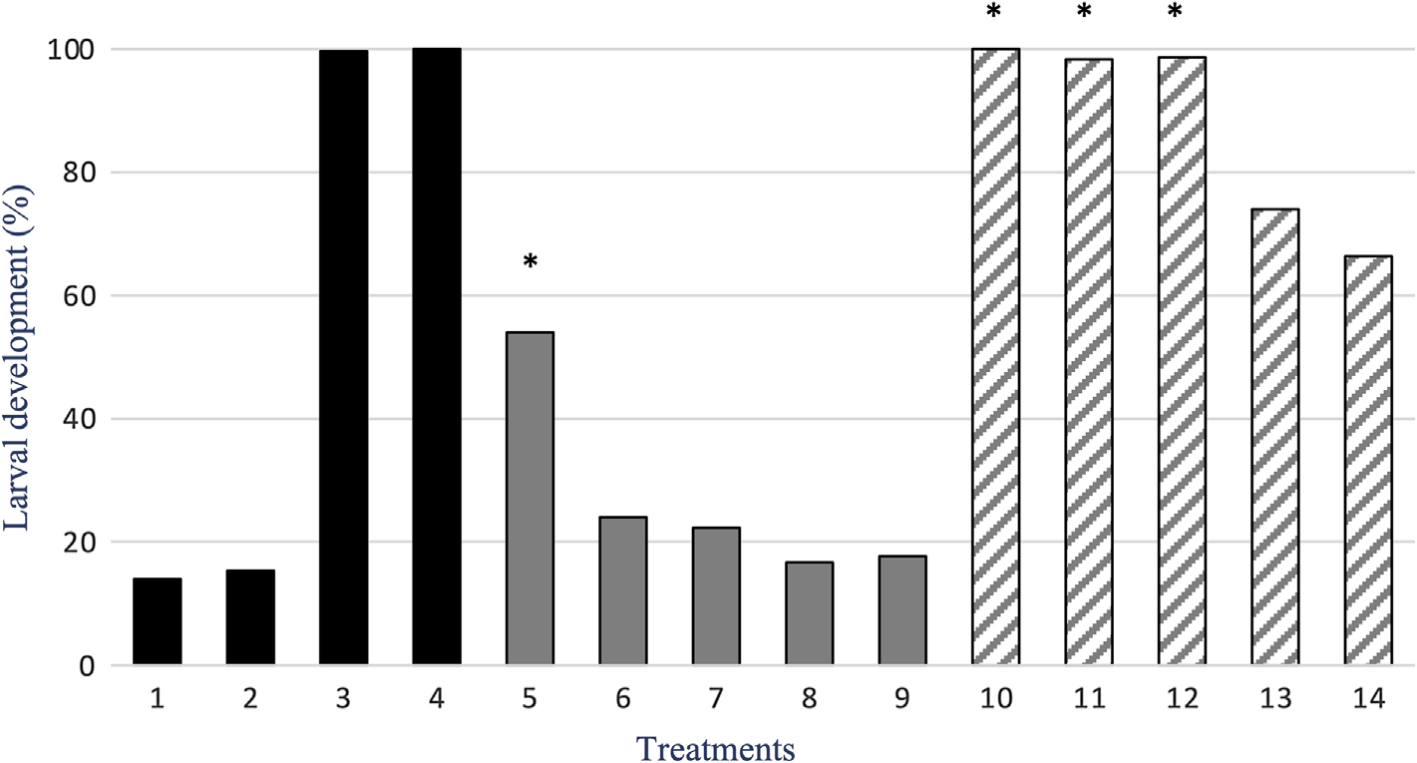
Percentage (%) of inhibition of gastrointestinal strongyle larval development by five concentrations of *Glycyrrhiza glabra* aqueous extract and of glycyrrhetinic acid. Columns: 1: Untreated, 2: DMSO, 3: Thiabendazole, 4: Thiabendazole plus DMSO, 5: 30 mg/mL, 6: 10 mg/mL, 7: 5 mg/mL, 8: 1 mg/mL, 9: 0.5 mg/mL of *G. glabra*, and 10: 30 mg/mL, 11: 10 mg/mL, 12: 5 mg/mL, 13: 1 mg/mL, 14: 0.5 mg/mL of glycyrrhetinic acid.

The results obtained with the LDT are shown in Figure 3. The untreated and the solvent DMSO control groups showed some small (14–19%) effects on L1 to L3 development, while TBZ and TBZ plus DMSO showed full efficacy also in this assay. In this test, only *G. glabra* showed a concentration-dependent effect with an *R*^2^ = 0.924. However, glycyrrhetinic acid had *R*^2^ = 0.910 with 64% efficacy also at lower concentrations. For *G. glabra*, the efficacy of the highest concentration (30 mg/mL) was significantly higher (*p* < 0.001) from that of all other concentrations. As for glycyrrhetinic acid, the efficacy of the three highest concentrations was significantly higher (*p* < 0.001) than that of the two lowest concentrations. Glycyrrhetinic acid showed significantly higher efficacy against L3 development when compared to *G. glabra*, by an average of 30%. For the LDT, the LC_50_ observed for glycyrrhetinic acid (5.10 mg/mL) was 2.7-fold lower, when compared to that of *G. glabra* (13.67 mg/mL) (Fig. 2b).

**Figure 4:**
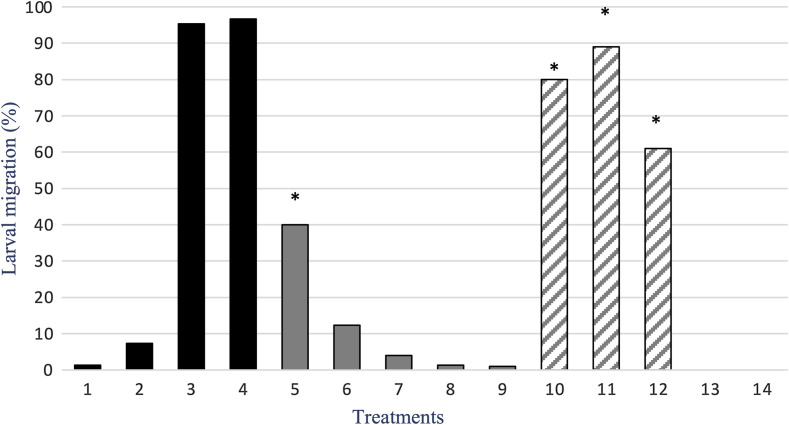
Percentage (%) of inhibition of gastrointestinal strongyle larval migration by five concentrations of *Glycyrrhiza glabra* aqueous extract and of glycyrrhetinic acid. Columns 1: Untreated, 2: DMSO, 3: Thiabendazole, 4: Thiabendazole plus DMSO, 5: 30 mg/mL, 6: 10 mg/mL, 7: 5 mg/mL, 8: 1 mg/mL, 9: 0.5 mg/mL of *G. glabra*, and 10: 30 mg/mL, 11: 10 mg/mL, 12: 5 mg/mL, 13: 1 mg/mL, 14: 0.5 mg/mL of glycyrrhetinic acid.

The data obtained in the LMIT are shown in Figure 4. The untreated and the solvent DMSO control groups showed only some small (1–7%) effects on L3 migration, while TBZ and TBZ plus DMSO had efficacies > 95%. *Glycyrrhiza glabra* showed a clear concentration-dependent effect with *R*^2^ = 0.971, while glycyrrhetinic acid had *R*^2^ = 0.855, with a drastic reduction of efficacy at lower concentrations. For *G. glabra*, the efficacy of the highest concentration (30 mg/mL) was significantly different (*p* < 0.001) with respect to all other concentrations. The efficacy of glycyrrhetinic acid at the three highest concentrations was significantly different (*p* < 0.001) from that of the two lowest concentrations. Moreover, glycyrrhetinic acid showed efficacy against L3 development > 400% higher on average when compared to that of *G. glabra*. For the LMIT, the LC_50_ of glycyrrhetinic acid (~5.12 mg/mL) was > 2.0-fold lower when compared to that of *G. glabra* (12.25 mg/mL) (Fig. 2c).

Concerning the cytotoxicity assay, results indicated a dose-dependent effect and low toxicity of *G. glabra* root extract. More specifically, 23.5%, 5.4%, 2.3%, 0.16% and 0.08% cytotoxicity were observed for 30, 10, 5, 1, and 0.5 mg/mL of the extract, respectively.

L3 identification showed that *Trichostrongylus* spp. (39.7%), *Teladorsagia/Ostertagia* spp. (19.2%), *Cooperia* spp. (14.3%), *Chabertia ovina* (7.4%), *Oesophagostomum* spp. (6.8%), *Bunostomum* spp. (1.8%), *Haemonchus* spp. (1%), and *Strongyloides papillosus* larvae (9.8%) were present in faecal pools used in the assays. Differential identification and counting of *Teladorsagia* spp. and *Trichostrongylus* spp. were performed based on morphology of ensheathed and exsheathed L3 according to the method reported by van Wyk and Mayhew [49]. The L3 of *S. papillosus* were distinguished from those of gastrointestinal strongyles based on their smaller width, oesophagus length (about 40% of the total body length), the bifid tip of the tail and the absence of sheath tail extension [49]. *Nematodirus* spp. eggs were not found at faecal microscopical examination.

## Discussion

The present study evaluated the *in vitro* ability of an aqueous extract of *G. glabra* roots containing 10% glycyrrhetinic acid (mainly as glycyrrhizinic acid saponin) and of pure glycyrrhetinic acid, against free-living stages (eggs and larvae) of a mixture of sheep GINs found in naturally infected sheep. Considering that on sheep farms it is unlikely that GIN infections are caused by a single nematode genus/species, in the *in vitro* assays we decided to use faecal samples collected from sheep naturally infected by different genera/species. However, this choice may also represent a limitation of the study since the *in vitro* anthelmintic activity of the two compounds examined here cannot be targeted to a specific sheep gastrointestinal nematode species. Nevertheless, considering that in the faecal samples used in this study most of these nematodes were found to belong to the genera *Trichostrongylus*, *Teladorsagia*/*Ostertagia* and *Cooperia*, which represented about the 75% of the totality of identified larvae, it can be assumed that the anthelmintic effects observed for *G. glabra* extract and glycyrrhetinic acid should be attributed at least to these genera.

*Glycyrrhiza* spp. plants, mainly *G. glabra*, glycyrrhizinic acid and glycyrrhetinic acid show several pharmacological properties *in vitro,* and in some case also *in vivo*, including antiviral (including HIV, SARS-Coronavirus, Hepatitis B, and C viruses, influenza A virus, porcine reproductive and respiratory syndrome virus, and rotavirus), antibacterial (*Bacillus subtilis*, *Staphylococcus aureus*, *S. epidermis*, *E. coli*, *Pseudomonas aeruginosa*, *Mycobacterium bovis*, *Helicobacter pylori*), antifungal (*Candida albicans*), and antiprotozoal (*Plasmodium falciparum*, *Leishmania donovani*) activities [3, 6, 7, 15–17, 21, 23, 30, 31, 38–41]. Moreover, the anthelmintic activity against *B. malayi*, *S. mansoni*, and *H. contortus* has also been demonstrated in previous studies [1, 27, 29]. For all these properties, glycyrrhizinic acid and glycyrrhetinic acid are considered scaffold molecules for the design and development of new bioactive compounds [20, 30].

In this study, both *G. glabra* root aqueous extract and glycyrrhetinic acid were found to be highly active when used against eggs (EHT), with a marked concentration-dependent effect. However, especially in the LDT and the LMIT, glycyrrhetinic acid showed significantly higher efficacy against sheep GINs compared to that of the plant extract. In LDT, glycyrrhetinic acid was found to be highly effective also at its lowest concentrations. Moreover, in the LDT and the LMIT, the anthelmintic activity of glycyrrhetinic acid at the highest concentration was comparable to that of TBZ. These results were also confirmed by the LC50 found for these two compounds in the different *in vitro* assays. In fact, while in the EHT similar LC50 values were observed for both candidates, in the LDT and LMIT, the LC50 of glycyrrhetinic acid was more than two fold lower than that of *G. glabra* extract. On the other hand, in this study the liquorice aqueous extract also showed valuable anthelmintic properties *in vitro* against GINs of sheep. In fact, the highest concentration of *G. glabra* extract (30 mg/mL) was able to significantly inhibit (>60%) the hatch of eggs and larval development from L1 to L3 of these nematodes when compared to the untreated controls, while its efficacy against L3 was lower.

These findings confirm, at least partially, the *in vitro* anthelmintic efficacy reported for the aqueous extract of *G. glabra* rhizome against adults of the sheep gastric nematode species *H. contortus* [29]. Although *G. glabra* aqueous extract tested in this study was obtained from the root of this plant, *in vitro* anthelmintic properties of *G. glabra* aqueous extract was found here also on eggs and larvae of sheep gastrointestinal nematodes. Obtained results also confirm the anthelmintic properties on sheep gastrointestinal nematodes previously reported for glycyrrhetinic acid on *B. malayi* microfilariae [27]. However, data from the evaluation of the Pearson coefficient suggest that the two compounds may have different mechanisms of action.

Glycyrrhetinic acid is considered the major active component of *G. glabra* root extract, which may contain about 2–25% glycyrrhetinic acid, both as such and as the glycosidic glycyrrhizinic acid saponin [23]. In humans, it is in fact known that after oral administration, glycyrrhizinic acid is hydrolysed to glycyrrhetinic acid mainly by the intestinal bacteria β-D-glucuronidase [20, 40]. Therefore, results obtained in this study suggest that the anthelmintic activity of *G. glabra* aqueous extract may rely mainly on its content in glycyrrhizinic acid/glycyrrhetinic acid, as the effect of the pure compound glycyrrhetinic acid was significantly higher than that of the extract. However, the possibility that other components normally present in the *G. glabra* aqueous extract, such as flavonoids, phytosterols and tannins [26], may also have contributed to its anthelmintic efficacy cannot be definitively ruled out.

Among the activities of glycyrrhetinic acid, this compound is proposed to strengthen enterocyte membrane integrity against both oxidative and proteolytic damage [2, 16, 41, 52]. Considering these protective properties on the intestinal epithelium and considering also that the production of glycyrrhetinic acid occur mainly in the intestine, it is possible to assume that *in vivo* experiments would demonstrate the potential anthelmintic activity and the beneficial effects on the gut of glycyrrhetinic acid, probably acting mostly on the GIN species infecting the intestinal tract of ruminants*.* On the other hand, it is also plausible to assume that glycyrrhizinic acid could act also on abomasal GIN species, like *T. axei*, *Teladorsagia* spp. and *H. contortus*. Therefore, further *in vivo* investigations on gastrointestinal strongyle-infected ruminants should include the evaluation of the anthelmintic efficacy of both glycyrrhizinic and glycyrrhetinic acids. Nevertheless, studies have found that GIN infection may alter gut function also by inducing considerable changes in the gut microbiome, for example in the case of GIN infecting the abomasum. This mechanism would involve lowering the acidic environment that functions as a potent barrier limiting the presence and growth of most bacteria [32]. These modifications can also result in several abomasal diseases in ruminants, frequently caused by *Clostridium* spp., a bacterial genus known to produce β-D-glucuronidase and that thus may be able to hydrolyse glycyrrhizinic acid contained in the *G. glabra* aqueous extract into glycyrrhetinic acid [48]. Although pharmacokinetic data for glycyrrhetinic acid in ruminants are not available, in rats the time for this compound to reach maximum plasma concentration is high and this implies slow intestinal absorption, probably also due to the slow conversion of glycyrrhizic acid to glycyrrhetinic acid in the intestine [53]. Moreover, glycyrrhizic acid shows positive pharmacokinetic properties in view of a possible use as an anthelmintic for the treatment of ruminants infected by gastrointestinal nematodes. In fact, although its maximum plasma concentration in rats is within five hours, this compound shows a reabsorption phenomenon by hepato-enteral circulation [53].

Concerning the toxicity of these compounds, results from this study and previous reported data seem to indicate that *G. glabra* aqueous root extract used in this study, glycyrrhizic and glycyrrhetinic acids may show low toxic effects. In fact, when used as a flavouring in food, glycyrrhizic acid at 1 mg/kg of complete feed is considered safe in all domestic animal species, including ruminants [12]. In humans, 100–200 mg/day is the upper limit considered safe for ingestion of glycyrrhizin, that correspond to less than 150 gr of liquorice (*G. glabra*) [20]. Moreover, results of the assay performed in this study showed low cytotoxicity for *G. glabra* aqueous root extract, as from 76.5% to about 100% cell viability was observed in ruminant cell lines following the treatment with the same concentrations (30, 10, 5, 1, and 0.5 mg/mL) tested in this study for evaluating the *in vitro* anthelmintic properties of the extract. Although glycyrrhetinic acid may block gap junction intracellular communication in a dose-dependent manner in animal and human cells, it is considered cytotoxic only at high concentrations, with cell viabilities from about 40–80% to over 90% observed in different human and animal tumoral cell lines treated with different concentrations of this compound [9, 10, 46]. Moreover, glycyrrhetinic acid and glycyrrhizic acid have anti-inflammatory effects in rats and mice and protect liver tissues [10]. Interestingly, liquorice ethanolic extract supplementation in the diet at 4.5% of dry matter was demonstrated to have a limited impact on sheep rumen function and improved sheep blood immunoglobulin level and anti-oxidative status [22]. In addition, like other saponins, glycyrrhizic acid has been considered able to modulate rumen bacteria, specifically to inhibit the growth of acetate-producing bacteria and to reduce the population of rumen protozoa. In sheep, these abilities have been associated with positive effects on the ruminal concentrations of total volatile fatty acids, acetate, propionate, and butyrate [22]. Moreover, a positive role of dietary *G. glabra* root supplementation on chemical and physical properties of cow milk and cheeses has recently been reported [4], as *G. glabra* root supplementation was found to be able to reduce lipid oxidation and induce positive changes in the colour and flavour of cow cheese.

In conclusion, *G. glabra* root aqueous extract and glycyrrhetinic acid showed favourable anthelmintic properties on GINs of sheep in all *in vitro* tests, although glycyrrhetinic acid was more effective than *G. glabra* root aqueous extract. However, *G. glabra* root aqueous extract showed low toxicity on ruminant cell lines when used at high concentrations. Further studies aimed at evaluating the *in vivo* efficacy of the liquorice (*G. glabra*) aqueous extract used in this study and of glycyrrhetinic acid as ecofriendly antiparasiticides on naturally infected sheep are encouraged.
